# Breviscapine alleviates myocardial ischemia-reperfusion injury in diabetes rats

**DOI:** 10.1590/acb390224

**Published:** 2024-02-26

**Authors:** Zhenhong Su, Yuanmei Zheng, Meng Han, Deqing Zhao, Zhi Huang, Yijun Zhou, Wenbing Hu

**Affiliations:** 1 Hubei Polytechnic University – Medical College – Hubei Key Laboratory for Kidney Disease Pathogenesis and Intervention – Huangshi, China.; 2 Affiliated Hospital of Hubei Polytechnic University – Huangshi Central Hospital – Huangshi, China.; 3 Zhejiang Chinese Medical University – Chinese Herbal Pieces Co. Ltd. – Quzhou, China.

**Keywords:** Myocardial, Ischemia-Reperfusion Injury, Diabetes Mellitus, Inflammatory Response, Oxidative Stress

## Abstract

**Purpose:**

To investigate the protective effect of breviscapine on myocardial ischemia-reperfusion injury (MIRI) in diabetes rats.

**Methods:**

Forty rats were divided into control, diabetes, MIRI of diabetes, and treatment groups. The MIRI of diabetes model was established in the latter two groups. Then, the treatment group was treated with 100 mg/kg breviscapine by intraperitoneal injection for 14 consecutive days.

**Results:**

After treatment, compared with MIRI of diabetes group, in treatment group the serum fasting blood glucose, fasting insulin, homeostasis model assessment of insulin resistance, and glycosylated hemoglobin levels decreased, the serum total cholesterol, triacylglycerol, and low-density lipoprotein cholesterol levels decreased, the serum high-density lipoprotein cholesterol level increased, the heart rate decreased, the mean arterial pressure, left ventricular ejection fraction, and fractional shortening increased, the serum cardiac troponin I, and creatine kinase-MB levels decreased, the myocardial tumor necrosis factor α and interleukin-6 levels decreased, the myocardial superoxide dismutase level increased, and the myocardial malondialdehyde level decreased (all P < 0.05).

**Conclusions:**

For treating MIRI of diabetes in rats, the breviscapine can reduce the blood glucose and lipid levels, improve the cardiac function, reduce the myocardial injury, and decrease the inflammatory response and oxidative stress, thus exerting the alleviating effect.

## Introduction

Diabetes is a chronic metabolic disease. In diabetes patients, the long-term stimulation of high glucose in body can cause the glucose and lipid metabolism disorder, oxidation and inflammatory reaction, and cardiac myocyte hypertrophy and fibrosis, leading to the cardiovascular diseases^1^. The risk of cardiovascular diseases of diabetes patients is higher than that of non-diabetes patients, which seriously endangers the health and life of patients. The myocardial ischemia injury is one of the main factors leading to the death of diabetes patients^2^. The commonly used treatment method for myocardial ischemia injury is to promptly restore the blood supply to the ischemic area. However, this may cause the more severe myocardial damage, namely, myocardial ischemia-reperfusion injury (MIRI)^3^. Therefore, exploring methods to alleviate the MIRI of diabetes patients is of great significance.

Breviscapine (C_21_H_18_O_12_) is a flavonoid extracted from the medicinal plant *Erigeron breviscapus* 4. The experimental study and clinical observation have found that the pharmacological actions of breviscapine include dilating micro-blood vessels, reducing blood viscosity and improving microcirculation^5^. Breviscapine can alleviate the ischemia-reperfusion injury of brain, liver, and other organs^6^^,^ 7. However, whether breviscapine has protective effect on MIRI of diabetes has not been reported.

In the present study, the MIRI model of diabetes rats was established, and the protective effect of breviscapine on MIRI of diabetes and the underlying mechanisms were investigated. The objective here was to provide an experimental reference for the clinical application of breviscapine to the treatment of MIRI of diabetes.

## Methods

This study was performed with the approval of ethics committee of Affiliated Hospital of Hubei Polytechnic University. All animal procedures followed the Guide for the Care and Use of Laboratory Animals by the National Institutes of Health.

### Establishment of myocardial ischemia-reperfusion injury of diabetes model of rats

Forty male Sprague Dawley rats (180–220 g; Shanghai SLAC Experimental Animal Co., Ltd., Shanghai, China) were randomly divided into control group, diabetes group, MIRI of diabetes group, and treatment group, with 10 rats in each group. The rats in control group were fed with common diet. The rats in other three groups were fed with the high-fat and high-sugar diet. The MIRI of diabetes model of rats was established according to the reported methods^8^^,^ 9, with some revisions. After feeding for 30 days, the rats in diabetes, MIRI of diabetes and treatment groups received the single sterile intraperitoneal injection of 30 mg/kg streptozotocin, and the rats in control group received the single sterile intraperitoneal injection of equal volume of citric acid-sodium citrate buffer. The injection was performed for five successive days. Finally, the blood was collected through the tail vein of rats. The fasting blood glucose (FBG) level ≥ 16.7 mmol/L indicated the diabetes model. In this study, the diabetes model was successfully established in 30 rats in diabetes, MIRI of diabetes and treatment groups.

In MIRI of diabetes and treatment groups, the rats were anesthetized with 3% pentobarbital sodium. Then, the trachea was separated and exposed, and the tracheal tube was inserted to connect the small-animal ventilator for mechanical ventilation. The thoracic cavity was opened, and the heart was exposed. The left anterior descending branch of coronary artery was ligated by silk thread for 30 min. The myocardial cyanosis combined with rise of ST segment in lead II by 0.1 mV or T-wave towering was the sign of successful ligation. After 30 min of ligation, the ligation thread was loosened, followed by reperfusion for 120 min. The ischemic myocardium turning red and the elevated ST segment or T wave falling back were the sign of successful reperfusion. In this study, the MIRI model was successfully established in 20 rats in MIRI of diabetes and treatment groups. In diabetes group, the silk thread crossed the surface layer of heart, but the coronary artery was not ligated, which lasted for 120 min.

### Treatment

After the successful establishment of the MIRI of diabetes model of rats, the rats in treatment group were given breviscapine (100 mg/kg; Hunan Hengsheng Pharmaceutical Co., Ltd., Hengyang, China) by intraperitoneal injection. The other three groups were given the same amount of normal saline by intraperitoneal injection. The treatment was performed once a day, for 14 consecutive days. No rat died during the treatment process.

### Cardiac function test

At the end of treatment, the rats were anesthetized with 3% pentobarbital sodium. The cardiac function indexes including heart rate (HR), mean arterial pressure (MAP), left ventricular ejection fraction (LVEF), and fractional shortening (FS) were tested using the small animal ultrasound imaging system.

### Determination of blood indexes

At the end of treatment, the blood of the abdominal aorta was taken, followed by centrifugation at 3,000 rpm for 10 min. The serum was obtained. The glucose metabolism indexes including FBG, fasting insulin (FINS), homeostasis model assessment of insulin resistance (HOMA-IR) and glycosylated hemoglobin (HbA1c) and lipid metabolism indexes including total cholesterol (TC), triacylglycerol (TG), high-density lipoprotein cholesterol (HDL-C) and low-density lipoprotein cholesterol (LDL-C) were detected using fully automatic biochemical analyzer. The myocardial injury indexes including cardiac troponin I (cTnI) and creatine kinase-MB (CK-MB) were determined by enzyme-linked immunosorbent assay.

### Determination of myocardial inflammatory response and oxidative stress indexes

After blood taking, the rats were sacrificed by cervical dislocation. The heart of rats was taken, and the 10% tissue homogenate was prepared in an ice bath. The inflammatory response indexes including tumor necrosis factor α (TNF-α) and interleukin-6 (IL-6) and oxidative stress indexes including superoxide dismutase (SOD) and malondialdehyde (MDA) were determined by enzyme-linked immunosorbent assay.

### Statistical analysis

Data were analyzed by Statistical Package for the Social Sciences 20.0 statistics software, and the measurement data were expressed as mean ± standard deviation. One-way analysis of variance was applied in the comparison among four groups. P < 0.05 was considered as significantly different.

## Results

### Comparison of glucose metabolism indexes among four groups

**Figure 1:**
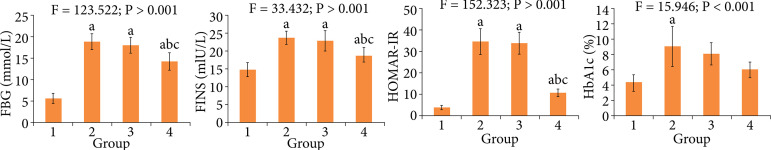
Comparison of glucose metabolism indexes among four groups.

Source: Elaborated by the authors. ^a^P < 0.05 vs. group 1; ^b^P < 0.05 vs. group 2; ^c^P < 0.05 vs. group 3; group 1: control; group 2: diabetes; group 3: myocardial ischemia-reperfusion injury of diabetes; group 4: treatment; FBG: fasting blood glucose; FINS: fasting insulin; HOMA-IR: homeostasis model assessment of insulin resistance; HbA1c: glycosylated hemoglobin.

At the end of the treatment, compared with control group, in diabetes, MIRI of diabetes and treatment groups the serum FBG, FINS, HOMA-IR and HbA1c levels were significantly increased, respectively (P < 0.05). Compared with diabetes and MIRI of diabetes groups, each index in treatment group was significantly decreased, respectively (P < 0.05) (Fig. 1).

### Comparison of lipid metabolism indexes among four groups

**Figure 2:**
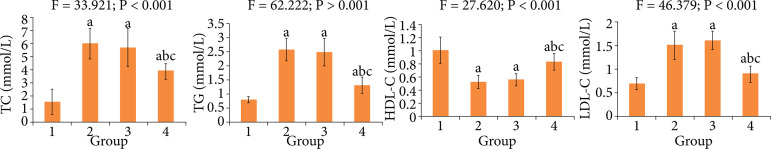
Comparison of lipid metabolism indexes among four groups.

Source: Elaborated by the authors. ^a^P < 0.05 vs. group 1; ^b^P < 0.05 vs. group 2; ^c^P < 0.05 vs. group 3; group 1: control; group 2: diabetes; group 3: myocardial ischemia-reperfusion injury of diabetes; group 4: treatment; TC: total cholesterol; TG: triacylglycerol; HDL-C: high-density lipoprotein cholesterol; LDL-C: low-density lipoprotein cholesterol.

As shown in Fig. 2, at the end of treatment the serum TC, TG and LDL-C levels in diabetes, MIRI of diabetes and treatment groups were significantly higher than those in control group, respectively (P < 0.05), and the serum HDL-C level in diabetes, MIRI of diabetes and treatment groups was significantly lower than that in control group, respectively (P < 0.05). Compared with diabetes and MIRI of diabetes groups, in treatment group the TC, TG and LDL-C levels were significantly decreased, respectively (P < 0.05), and the HDL-C level was significantly increased, respectively (P < 0.05).

### Comparison of cardiac function indexes among four groups

**Figure 3:**
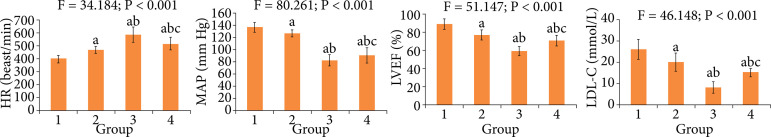
Comparison of cardiac function indexes among four groups.

Source: Elaborated by the authors. ^a^P < 0.05 vs. group 1; ^b^P < 0.05 vs. group 2; ^c^P < 0.05 vs. group 3; group 1: control; group 2: diabetes; group 3: myocardial ischemia-reperfusion injury of diabetes; group 4: treatment; HR: hear rate; MAP: mean arterial pressure; LVEF: left ventricular ejection fraction; FS: fractional shortening.

At the end of treatment, compared with control group, in diabetes, MIRI of diabetes and treatment groups the HR was significantly increased, respectively (P < 0.05), and the MAP, LVEF and FS were significantly decreased, respectively (P < 0.05). Compared with diabetes and MIRI of diabetes groups, in treatment group the HR was significantly decreased, respectively (P < 0.05), and the MAP, LVEF and FS were significantly increased, respectively (P < 0.05) (Fig. 3).

### Comparison of myocardial injury indexes among four groups

**Figure 4:**
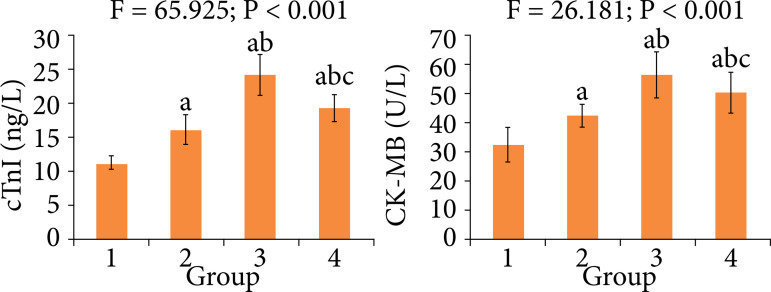
Comparison of myocardial injury indexes among four groups.

Source: Elaborated by the authors. ^a^P < 0.05 vs. group 1; ^b^P < 0.05 vs. group 2; ^c^P < 0.05 vs. group 3; group 1: control; group 2: diabetes; group 3: myocardial ischemia-reperfusion injury of diabetes; group 4: treatment; cTnI: cardiac troponin I; CK-MB: creatine kinase-MB.

Figure 4 showed that, at the end of treatment, the serum cTnI and CK-MB levels in diabetes, MIRI of diabetes and treatment groups were significantly higher than those in control group, respectively (P < 0.05). Compared with diabetes and MIRI of diabetes groups, each index in treatment group was significantly decreased, respectively (P < 0.05).

### Comparison of inflammatory response indexes among four groups

**Figure 5:**
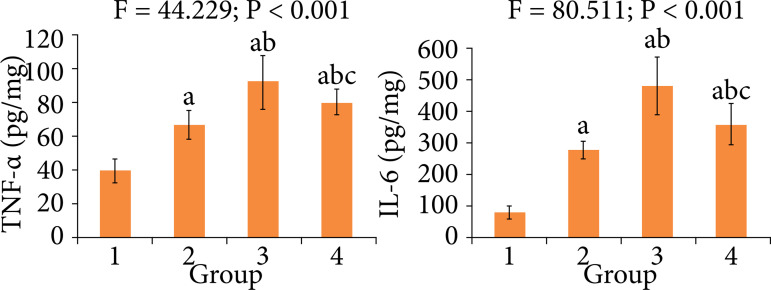
Comparison of inflammatory response indexes among four groups.

Source: Elaborated by the authors. ^a^P < 0.05 vs. group 1; ^b^P < 0.05 vs. group 2; ^c^P < 0.05 vs. group 3; group 1: control; group 2: diabetes; group 3: myocardial ischemia-reperfusion injury of diabetes; group 4: treatment; TNF-α: tumor necrosis factor α; IL-6: interleukin-6.

At the end of treatment, compared with control group, in diabetes, MIRI of diabetes and treatment groups the myocardial TNF-α and IL-6 levels were significantly increased, respectively (P < 0.05). Compared with diabetes and MIRI of diabetes groups, each index in treatment group was significantly decreased, respectively (P < 0.05) (Fig. 5).

### Comparison of oxidative stress indexes among four groups

**Figure 6:**
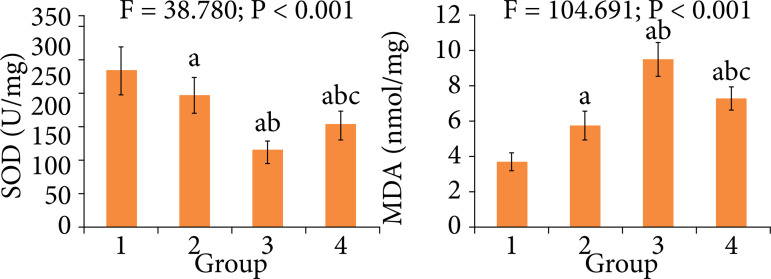
Comparison of oxidative stress indexes among four groups.

Source: Elaborated by the authors. ^a^P < 0.05 vs. group 1; ^b^P < 0.05 vs. group 2; ^c^P < 0.05 vs. group 3; group 1: control; group 2: diabetes; group 3: myocardial ischemia-reperfusion injury of diabetes; group 4: treatment; SOD: superoxide dismutase; MDA: malondialdehyde.

As shown in Fig. 6, at the end of treatment the myocardial SOD level in diabetes, MIRI of diabetes and treatment groups was significantly lower than that in control group, respectively (P < 0.05), and the myocardial SOD level in diabetes, MIRI of diabetes and treatment groups was significantly higher than that in control group, respectively (P < 0.05). Compared with diabetes and MIRI of diabetes groups, in treatment group the SOD level was significantly increased, respectively (P < 0.05), and the MDA level was significantly decreased, respectively (P < 0.05).

## Discussion

With the gradual acceleration of aging, the number of patients with diabetes is increasing year by year, which seriously threatens people’s health. Diabetes is an independent risk factor of ischemic heart disease. It aggravates the MIRI and the main cause of death of diabetes patients^10^. Finding drugs that can effectively reduce the MIRI of diabetes is one of the focuses of the current research.

In the present study, the MIRI model of diabetes rats was established, and the protective effect of breviscapine on MIRI of diabetes were investigated. Results showed that, at the end of treatment, compared with MIRI of diabetes group, in treatment group the glucose metabolism indexes, lipid metabolism indexes and cardiac function indexes were significantly improved. This suggests the breviscapine treatment can improve the glucose and lipid metabolism, and alleviate the cardiac function in rats with MIRI of diabetes. Wang et al.^11^ have found that breviscapine can ameliorate the cardiac dysfunction in streptozotocin-induced diabetic rats, and the results obtained here are similar.

During MIRI, the myocardial cells degenerate, with necrosis. Under this condition, the myocardial cell membrane permeability increases, so the levels of myocardial injury markers in blood increases^12^. Then, the degree of myocardial injury is related to the levels of cTnI and CK-MB^13^. cTnI is a biomarker of myocardial injury, and a preferred blood test target for patients with acute myocardial infarction^14^. CK-MB is also a commonly used and valuable biochemical indicator for diagnosing acute myocardial injury. It reflects the extent of myocardial ischemia. The higher the serum CK-MB content, the more severe the degree of myocardial injury^15^. It is shown that breviscapine can prevent the myocardial injury^16^. Results of this study showed that, at the end of treatment, compared with MIRI of diabetes group, the serum cTnI and CK-MB levels in treatment group were significantly decreased. This indicates that the breviscapine treatment can reduce the myocardial injury for MIRI of diabetes rats.

TNF-α is the main mediator of inflammatory cascade reaction, which can cause the systemic inflammatory response^17^. The content of TNF-α is very lower in normal myocardial tissue. After MIRI, TNF-α is released by macrophages, monocyte, etc. in a few minutes, so the myocardial TNF-α level is increased. Inflammatory response is an important mechanism of MIRI^18^. IL-6 is a lymphokine produced by activated T cells and fibroblasts. It is an important member of the cytokine network and plays a central role in acute inflammatory response. IL-6 can mediate the acute phase response of the liver and stimulate the production of C-reactive protein and fibrinogen^19^. It was found that breviscapine can reduce the myocardial inflammation in the animal model of cardiovascular disease^20^. In our study, at the end of treatment, compared with MIRI of diabetes group, the myocardial TNF-α and IL-6 levels in treatment group were significantly decreased. This indicates that the breviscapine treatment can decrease the inflammatory response, thus alleviating the MIRI of diabetes in rats.

Oxidative stress plays an important role in tissue injury. The excessive production of oxygen free radicals and the imbalance of tissue scavenging ability of oxygen free radicals are the important causes of MIRI^21^. SOD is an important antioxidant enzyme in the body. It can catalyze the transformation of oxygen free radicals to hydrogen peroxide, thus avoiding the damage to cells^22^. MDA is a product of lipid peroxidation reaction of oxygen free radicals with cell membranes. The changes of MDA content can reflect the degree of injury caused by oxygen free radicals^23^. Liu et al.^24^ have found that breviscapine can block CCl_4_-induced oxidative stress by through improving anti-oxidants and impeding mitogen-activated protein kinase pathways. Results of this study showed that, at the end of treatment, compared with MIRI of diabetes group, in treatment group the myocardial SOD level was significantly increased, and the MDA level was significantly decreased. This indicates that the breviscapine treatment can enhance the body’s antioxidant capacity and decrease the oxidative stress, thus playing a protection role for MIRI of diabetes.

## Conclusions

In conclusion, for treating MIRI of diabetes in rats, the breviscapine can reduce the blood glucose and lipid levels, improve the cardiac function, reduce the myocardial injury, and decrease the inflammatory response and oxidative stress, thus exerting the alleviating effect. The research results can preliminarily demonstrate the protective effect of breviscapine on MIRI in diabetes rats, and provide a reference for clinical application of breviscapine to treatment of MIRI of diabetes.

This study still has some limitations. Firstly, we have not made the histological and/or immunohistochemistry analysis. Secondly, the other action mechanisms have not been investigated. These issues should be solved in next studies to make the findings more convincing.

## Data Availability

Data will be available upon request.
